# Development of the Japanese version of an information aid to provide accurate information on prognosis to patients with advanced non–small-cell lung cancer receiving chemotherapy: a pilot study

**DOI:** 10.1186/s12904-018-0292-6

**Published:** 2018-02-27

**Authors:** Kikuo Nakano, Yoshihiro Kitahara, Mineyo Mito, Misato Seno, Shoji Sunada

**Affiliations:** 1 Department of Respiratory Medicine, National Hospital Organization, Kure Medical Center, 3-1 Aoyama, Kure, Hiroshima, 737-0023 Japan; 2 Department of Palliative Medicine, National Hospital Organization, Kure Medical Center, 3-1 Aoyama, Kure, Hiroshima, 737-0023 Japan

**Keywords:** Information aid, Explicit prognostic information, Hope, Psychosocial disorder, Non-small-cell lung cancer, Chemotherapy

## Abstract

**Background:**

Without explicit prognostic information, patients may overestimate their life expectancy and make poor choices at the end of life. We sought to design the Japanese version of an information aid (IA) to provide accurate information on prognosis to patients with advanced non–small-cell lung cancer (NSCLC) and to assess the effects of the IA on hope, psychosocial status, and perception of curability.

**Methods:**

We developed the Japanese version of an IA, which provided information on survival and cure rates as well as numerical survival estimates for patients with metastatic NSCLC receiving first-line chemotherapy. We then assessed the pre- and post-intervention effects of the IA on hope, anxiety, and perception of curability and treatment benefits.

**Results:**

A total of 20 (95%) of 21 patients (65% male; median age, 72 years) completed the IA pilot test. Based on the results, scores on the Distress and Impact Thermometer screening tool for adjustment disorders and major depression tended to decrease (from 4.5 to 2.5; *P* = 0.204), whereas no significant changes were seen in scores for anxiety on the Japanese version of the Support Team Assessment Schedule or in scores on the Hearth Hope Index (from 41.9 to 41.5; *p* = 0.204). The majority of the patients (16/20, 80%) had high expectations regarding the curative effects of chemotherapy.

**Conclusion:**

The Japanese version of the IA appeared to help patients with NSCLC maintain hope, and did not increase their anxiety when they were given explicit prognostic information; however, the IA did not appear to help such patients understand the goal of chemotherapy. Further research is needed to test the findings in a larger sample and measure the outcomes of explicit prognostic information on hope, psychological status, and perception of curability.

**Electronic supplementary material:**

The online version of this article (10.1186/s12904-018-0292-6) contains supplementary material, which is available to authorized users.

## Background

Lung cancer is the most common cause of cancer-related death in males [1]. Approximately 85% of cancerous tumors are histological subtypes of non–small-cell lung cancer (NSCLC), and 50% of patients with NSCLC are treated with palliative chemotherapy [2]. In recent years, therapies have been developed to target aberrant oncogenic pathways. Epidermal growth factor receptor (EGFR) inhibitors have been shown to improve median overall survival; and survival for patients treated with gefitinib, platinum, and pemetrexed or docetaxel is around 3 years [3]. However, in NSCLC, the overall frequency of EGFR mutations is only around 30% [4]. Platinum-based chemotherapy may provide an improved quality of life with an absolute increase of approximately 1.5 months in median survival [5].

Without explicit prognostic information, patients may overestimate their life expectancy and make poor choices at the end of life [6, 7]. Truth-telling about life expectancy, which reflects modern Anglo-American values, is considered a requirement for patients’ self-determination [8]. This suggests the presence of major cross-cultural differences in truth-telling attitudes and practices [9]. Japan had had a long-standing tradition of paternalism with regard to patient-physician encounter in the decision-making process [10]. However, since the 2000s, informed consent and truth-telling attitudes have evolved in many countries where self-determination had been synonymous with isolation [9].

In practice, oncologists in the United States are often reluctant to give people poor prognostic information [11] and tend to avoid such discussions for fear of diminishing patient hope [12]. According to one model in the context of potential illness, hope is defined through two related spheres: a particular type of hope with expectations of a particular outcome, and a generalized type of hope that implies an absolute trust in the future without any specific predetermined goals [13]. In some cases, particular hopes such as an expectation of symptom alleviation resulting from chemotherapy appear unrealistic and misguided, leading patients to overestimate their life expectancy and to have a continuing belief that they will be cured.

One strategy to promote patient understanding and facilitate decision-making is the use of an information aid (IA). In the context of an IA, the discussion about life expectancy is one of the most important parts; however, it remains unclear whether numerical data or qualitative words are preferable in such discussions [14, 15]. The balance between truth-telling and maintaining hope is a delicate one [13]. To further investigate this issue and determine if patients would accept information on life expectancy in the form of numerical data, we designed a Japanese version of an IA for patients with metastatic NSCLC. We then assessed the number of patients who opt for full disclosure of life expectancy when using the IA, and the pre- and post-intervention effects of the IA on psychosocial disorder, hope, and perception of prognosis and treatment goals.

## Methods

### Development of the Japanese version of a patient IA about life expectancy

The IA was designed as a three-page pamphlet printed on letter-size paper. The content on the first and second pages included chemotherapy-related information on diagnosis, treatment goals, toxicity, regimens and schedules, and treatment options from the beginning of illness to the end of life. The third page [see Additional file 1] was composed of survival data from the Decision Aid for first-line cytotoxic chemotherapy as recommended by the American Society of Clinical Oncology [16], the Four-Arm Cooperative Study in Japan [17], and the NEJ002 trials [3]. In addition, estimates for average, best-, and worst-case life expectancy scenarios were provided for patients starting first-line chemotherapy [18].

### Patient selection

We had 69 patients who were diagnosed as incurable or metastatic NSCLC in our hospital during the survey. Thirty-two of those patients decided to undergo the first-line chemotherapy. In addition to carrying out visits by a palliative care team, we conducted a pilot test of the IA for life expectancy on 21 inpatients recruited through the National Hospital Organization Kure Medical Center from July 2013 to July 2014. Inclusion criteria were as follows: 1) diagnosis of incurable or metastatic NSCLC; 2) age 18 years or older; and 3) the decision to receive first-line chemotherapy had already been made. Patients were excluded if they were experiencing substantial distress or had marked psychiatric problems, or cognitive dysfunction.

### Measurements

#### Primary outcome

The primary outcome measures were the number of patients who would opt for explicit prognostic information, the impact of explicit prognostic information on hope as measured by the Hearth Hope Index (HHI) [19], and the impact of explicit prognostic information on psychosocial disorders as measured by both the Distress and Impact Thermometer (DIT) [20], a screening tool for adjustment disorders and/or major depression that has cut-off scores of “4” for “distress” and “3” for “impact”, and the Japanese version of the Support Team Assessment Schedule (STAS-J) [21]. The primary outcome measures of the STAS-J items were the patient anxiety items.

#### Secondary outcomes

The secondary outcomes measures included the expectations of patients about the effectiveness of chemotherapy after the interventions, which was calculated based on patient responses to an item adapted and modified from that used in a study by Weeks et al. [22]: “After talking with your doctors about chemotherapy, how likely did you think it was that chemotherapy would…help you live longer, cure your cancer, or help you with problems you were having because of your cancer?” Response options were “very likely,” “somewhat likely,” “a little likely,” “not at all likely,” and “don’t know.”

### Procedures

Interviews that included screening questions from the DIT and the HHI were also conducted within 2 weeks before the first-line chemotherapy was started after the diagnosis. The IA and interviews were administered by each patient’s attending physician and a palliative care team consisting of a psycho-oncologist, an oncology nurse, and an oncology pharmacist. Participants completed baseline questionnaires before being informed of the content on the first and second pages of the IA. Next, but before providing the survival data on the third page, we asked each patient if they were willing to view and listen to survival statistics. If they were not, the interview was complete. We emphasized that the survival data were in the form of statistics, and therefore could not specifically extrapolate to each particular individual’s life expectancies. Subsequently, the patients were assessed using the STAS-J three times within the next week by the oncology nurse of the palliative care teams. Post-intervention questionnaires regarding the effects of chemotherapy were conducted 3 weeks after post-baseline interviews.

### Statistical analysis

The paired data testing was conducted using related samples with Wilcoxon signed-rank test. Statistical significance was set at *p* = 0.05. All statistical analyses were performed using SPSS 17 (IBM Corp.).

### Ethical considerations

This study was conducted at a single hospital in Kure, Japan and approved by the institutional research ethics board. All study participants provided written informed consent to participate.

## Results

### Participants

As shown in Table 1, the median age of the participants was 72 years (range, 50–79 years), 65% were male, 90% had a performance status of 0 or 1, 70% had adenocarcinoma histology and 95% were at stage IV. A total of 16 patients received platinum-containing regimens, and four patients received dual tyrosine kinase and anaplastic lymphoma kinase inhibitors as first-line therapy. All had a STAS-J score 1 for anxiety.

**Table 1:** Patient characteristics

Characteristic	Number (%)
	*n* = 20
Age (years)	
Median	72
Range	50–79
Gender	
Male	13 (65)
ECOG performance status	
0	8 (40)
1	10 50)
2	2 (10)
Histology	
Adenocarcinoma	13 (65)
Squamous and other	7 (35)
EGFR mutation / ALK rearrangement	4 (20) / 1 (5)
First-line chemotherapy	
Platinum-based doublet	16 (80)
Targeted agents	4 (20)
Support Team Assessment Schedule scores	
Cough (more than 1)	11 (55)
Dyspnea (more than 1)	2 (10)
Pain (1)	7 (35)
Fatigue (more than 1)	4 (20)
Sleep disturbance (more than 1)	4 (20)
Patients’ anxiety (1)	20 (100)
Patients’ insight of advanced disease (0)	3 (15)

### Primary outcome

A total of 21 patients with NSCLC participated in the IA pilot test. One patient chose not to complete the test after starting due to refusal to listen to life expectancy statistics.

**Fig. 1:**
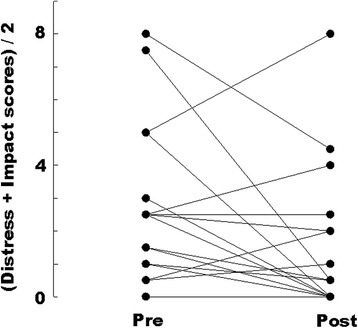
Pre- and post-intervention sore on the Distress and Impact Thermometer

The mean pre-intervention score on the DIT for “distress” and “impact” was 4.5 (standard deviation [SD] 5.7), and the mean post-intervention score was 2.5 (SD 6.5; *P* = 0.204, Wilcoxon signed-rank test) (Fig. 1). Four patients scored higher than 4 for “distress” and higher than 3 for “impact” at baseline on the DIT. However, after the discussion about life expectancy, this number decreased to one. No changes were seen in STAS-J scores for cough, dyspnea, and pain from the first visit of the palliative care team to 3 weeks later. In addition, no changes were seen in STAS-J scores for anxiety or insights regarding advanced disease (Table 2). The mean pre- and post-intervention scores on the HHI were 41.9 (SD 5.7) and 41.5 (SD 6.5), respectively (*P* = 0.204, Wilcoxon signed-rank test) (Table 2).

**Table 2:** Changes of Hearth Hope Index scores and Support Team Assessment Schedule scores pre- and post-intervention, and perception of prognosis and treatment goals

Patient	Hearth Hope Index scores		Support Team Assessment Schedule scores for anxiety		Perception of prognosis and treatment goals		
	Pre	Post	Pre	Post	Cure	Life expectancy	Alleviation
1	48	48	1	1	very lilely	very lilely	very lilely
2	39	41	1	1	a little likely	somewhat likely	somewhat likely
3	43	40	1	1	somewhat likely	somewhat likely	somewhat likely
4	36	39	1	1	a little likely	somewhat likely	somewhat likely
5	39	38	1	1	not at all likely	don’t know	somewhat likely
6	43	46	1	1	somewhat likely	somewhat likely	somewhat likely
7	46	46	1	1	very lilely	somewhat likely	very lilely
8	23	18	1	1	not at all likely	a little likely	not at all likely
9	44	40	1	1	somewhat likely	somewhat likely	somewhat likely
10	37	39	1	1	not at all likely	somewhat likely	somewhat likely
11	38	40	1	1	don’t know	somewhat likely	somewhat likely
12	45	38	1	1	somewhat likely	somewhat likely	somewhat likely
13	45	45	1	1	somewhat likely	somewhat likely	a little likely
14	46	45	1	1	somewhat likely	somewhat likely	don’t know
15	44	48	1	1	very lilely	very lilely	very lilely
16	45	41	1	1	somewhat likely	a little likely	somewhat likely
17	41	42	1	1	somewhat likely	somewhat likely	somewhat likely
18	45	44	1	1	somewhat likely	don’t know	somewhat likely
19	43	44	1	1	somewhat likely	don’t know	somewhat likely
20	48	48	1	1	very lilely	very lilely	very lilely
Mean scores	41.9	41.5					
SD	5.7	6.5					
	P = 0.204						

### Secondary outcomes

Overall, 80% of the patients gave answers that were not consistent with an understanding that chemotherapy was not at all likely to cure their cancer (Table 2).

## Discussion

The most successful IAs available for metastatic, incurable diseases effectively promote or sustain the hope of patients. Our findings suggest that IAs that provide prognostic information with explicit numerical data can sustain generalized hope in patients with advanced cancer without increasing their anxiety. Similarly, Smith et al. [23] reported that hope can be maintained in patients with advanced cancer when they are given truthful prognoses and treatment information. In a study involving 126 women with platinum-resistant ovarian cancer [24], generalized hope, but not particular hopes such as an expectation of symptom alleviation resulting from chemotherapy, was negatively correlated with anxiety and depression. Therefore, rather than avoiding discussions regarding life expectancy, oncologists should aim to provide information that is realistic, but also conveys hope. In the present study, the tendency in the DT scores after the discussion about life expectancy may be explained as chemotherapy providing a helpful role in the ability of some patients to adjust psychologically to their prognosis, or that the IA is an acceptable and highly informative tool.

Because a majority of patients seem to prefer to be left at least partially ignorant about life expectancy [14, 25], the importance of determining patients’ preferences before discussing prognoses should be emphasized [26, 27]. This preference demonstrates major cross-cultural differences; people in non-Anglo Saxon countries are less likely than Anglo Saxon countries to believe that a patient should be told about a terminal prognosis [28]. In Anglo Saxon countries, this knowledge is considered necessary for patients’ self-determination. Therefore, in Japan, where patients’ self-determination is more synonymous with isolation, it is speculated that truth-telling in discussions regarding prognosis should be avoided. To the best of our knowledge, this pilot study is the first attempt to develop and test an IA for patients with metastatic NSCLC in Japan. We found that 20 of 21 patients accepted prognostic information in the form of numerical data and were able to complete the IA test and interviews. The reason behind this high completion rate was thought to be the growing importance of informed consent, public knowledge about the nature of and treatments for cancer, and patient and public activism, regardless of cross-cultural differences. In this study, information about life expectancy was explained as follows [see Additional file 1]: “The numbers provided here refer to the outcomes of an average patient with this disease in this situation. Half of patients will perform better than this number and half will perform worse. Remember, you are not a statistic and therefore will not always match this number. Each person has different factors that may affect their response.” These statements seem to have resulted in the high rate of acceptance among patients regarding life expectancy. Another possible reason for the high completion of intervention using the IA is interviewer bias. It has been reported that physician and patient seem to mutually reinforce attitudes of not giving up on treatment in order to maintain patient hope [29]. Thus, our patients may have been eager to participate in our interviews as it touched upon starting chemotherapy.

Whether numerical data or qualitative words are preferable when discussing life expectancy with patients with incurable cancer remains unclear. Kaplowitz et al. [30] reported that less than half of the patients in their study wanted a quantitative estimate of survival. Meanwhile, Hagerty et al. [14] reported that a majority of the patients wanted detailed prognostic information. Recently, Kiely et al. [31] reported that providing numerical estimates for average, best-, and worst-case life expectancy scenarios would be reassuring and conveys hope to patients. Our findings were consistent with those of Kiely et al. However, because changes in a disease may alter a patient’s preferences for information about prognosis and treatment [32], hope among patients receiving second-line chemotherapy or beyond requires further investigation.

Even when presented with accurate information on prognosis and the goals of cancer treatment, patients with advanced cancer frequently retain inaccurate perceptions about their illness. A major reason for this prognostic misunderstanding is collusion between patients and their physicians involving quick transitions by both parties from discussions regarding prognosis to those regarding treatment options and schedules; this collusion misdirects attention and can lead to false optimism [22]. In order to avoid this situation, we provided explicit prognostic descriptions to patients while using the IA. However, we still found that 80% of the patients provided survey responses that indicated inaccurate expectations about the curative potential of chemotherapy. It is unclear whether this represents collusion, a misunderstanding of the IA, or misunderstanding of the question asked. The rate of these inaccurate responses was higher than those reported by Weeks et al. [22], and was consistent with a report by Leighl et al. [33] that used a treatment decision aid. Moreover, despite having terminal NSCLC, 32% of the patients in the study by Temel et al. [34] reported that their cancer was curable, and 69% expressed the idea that the goal of chemotherapy was to eliminate the cancer entirely. We guess that such conflicting views may be due to confusion about the nature of the chemotherapy, in which patients understand that their cancer is incurable, but nonetheless hope that curative therapy could be developed in the near future, or even that a miracle could occur.

Early palliative care has improved quality of life, mood, and survival in patients with metastatic NSCLC based on results from a randomized trial [35]. A qualitative analysis of that trial emphasized that palliative care clinicians focus on determining a patient’s individual needs and preferences for prognostic information in the early stages of the illness, and these palliative care clinicians play a distinct yet complementary role that enables oncologists to focus on cancer treatment and manage medical complications later in the trajectory of the disease [36]. The present study showed that only 15% of the patients demonstrated an accurate understanding of their illness; and no improvements in this understanding were seen 3 weeks after the intervention by palliative care teams. This suggests that palliative care teams could not effectively establish relationships with patients due to the traditional paternalistic relationship between patients and their physicians. Results from a recent trial suggest that a combination of explicit prognostic information and reassurance about non-abandonment might provide realistic hope that improved understanding can still be achieved [37]. This method is available for oncologists in Japan via a system in which attending physicians treat patients with powerful emotional expressions throughout the course of their disease. However, further research is needed to explore the complementary roles of palliative care teams and oncologists in Japan.

This study has several limitations. First, the evaluations were at a single center and comprised a small sample size. These findings need to be interpreted with caution. However, data regarding hope and anxiety was similar to past studies [23, 33]. Second, we did not examine longitudinal changes in psychological distress and hope. As it has been reported that longitudinal changes in depression symptoms are associated with increased mortality [38], the IA should be tested at the time of diagnosis and at the time of disease progression after first-line chemotherapy. Third, because the present study did not design trajectories of perception of prognosis and treatment goals before and after interviews, we could not evaluate this aspect. However, there have been reports that many patients hold inaccurate perceptions about their prognosis over time even with the provision of adequate information. Fourth, the IA lacked information regarding the sustained long survival achieved through the use of immune checkpoint inhibitors [39]. Development of a future IA with that new survival information is necessary, and it should be examined in a larger sample.

## Conclusion

The Japanese version of an IA appeared to help patients with NSCLC maintain hope, and did not increase their anxiety when they were given explicit prognostic information; however, the IA did not appear to help such patients understand the goal of chemotherapy. Further research is needed to test the findings in a larger sample and measure the outcomes of explicit prognostic information on hope, psychological status, and perception of curability.

## Additional file

Additional file 1: Information Aid for First-line Chemotherapy. (DOCX 42 kb)

## Abbreviations

DIT: Distress and Impact Thermometer
EGFR: Epidermal growth factor receptor
HHI: Hearth Hope Index
IA: Information aid
NEJ: North East Japan
NSCLC: Non–small-cell lung cancer
STAS-J: Japanese version of the Support Team Assessment Schedule
