# Supplementary material for: Development of the Japanese version of an information aid to provide accurate information on prognosis to patients with advanced non–small-cell lung cancer receiving chemotherapy: a pilot study
Source: BMC Palliat Care. 2018 Feb 27;17:38. doi: 10.1186/s12904-018-0292-6 (PMC5828180; doi:10.1186/s12904-018-0292-6)
Supplement: Supplementary file 1 — Information Aid for First-line Chemotherapy. (DOCX 42 kb) [file 12904_2018_292_MOESM1_ESM.docx]

**Information Aid**

**Inoperable Stage 3B or 4**

**Non-Small Cell Lung Cancer**

**First-line Chemotherapy**

1. Pathologic diagnosis

1) Adenocarcinoma

　2) Squamous cell carcinoma

　3) Other

2. Clinical evaluation

　1) Clinical stage inoperable Stage 3B or 4

2) Performance status

3) Other

3. Treatment recommendations

1) Chemotherapy

2) Palliative radiation therapy

3) Opioids for palliative care

4) Palliative care services with the palliative care team

4. The goal of chemotherapy

1) The longest life

　2) The best quality of life

　3) The fewest side effects

5. Chemotherapy regimens and duration

　1) Cytotoxic agents

2) Targeted agents

・If your cancer is shrinking in the first month after initiation, chemotherapy will be continued for a long time.

・If therapy is not well tolerated, then stopping it is reasonable, even if your cancer has responded to it.

・At reassessment after a set period of time, if second or third-line of chemotherapy is meeting the goal with acceptable results, it will be continued.

6. Side effects

The most common side effects will vary with the type of treatment given. Some of side effects that

may require stopping, delaying, or lowering the dose of your chemotherapy include the following:

Side Effect 　　 How likely ? (%) 　　　Additional Facts

Hair loss 　 　12 ~ 78 　　　　 Almost all patients experience

　　　　　 some hair loss, reversible

Mouth sores 　　　 2 　　　 Symptom relief possible

Nausea with or 　　 4 ~ 10 　　　 Medicine usually can help

without vomiting

Appetite loss 　 17 ~ 27 Reversible

Diarrhea 6　　　　　　　　 Medicine usually can help

Fatigue 5 ~ 7 Reversible

Low white blood cell 1 ~ 7 Reversible, treatable, but potentially

count and infection life threatening

Low platelet count 1 ~ 19 May or may not result in bleeding;

treatable by transfusion

Anemia 14 ~ 19 Reversible, treatable by transfusion

Interstitial lung 6 Treatable, but potentially life threatening

disease

Skin rash 20 ~ 40 Medicine usually can help

Chemotherapy-related 1

death

7. Shift from chemotherapy to hospice

As subsequent regimens become less beneficial and more toxic, we will discuss discontinuing chemotherapy and transitioning to hospice care.

8. Life expectancy

The numbers provided here refer to the outcomes of the average patient with this disease in this situation. Half of all patients will perform better than this number and half will perform worse. Remember that you are not a statistic and therefore will not always match this number. Each person has different factors that may affect their response.

1) The chance of being alive at one year

The following figure provides some estimates from a recent study. The chance of being alive at one year will increase with chemotherapy.

2) The chance of being cured by chemotherapy

In this setting, there is no chance of cure. The goal is to control the disease and any symptoms for as long as possible.

3) Three scenarios for survival

The typical person with your type and stage of cancer lives for X months.

Average-case scenario: About half of the people with your type and stage of cancer live between X/2 and 2X months.

Best-case scenario: If 100 people had exactly the same type and stage of cancer as you, the 10 who did the best would　still be alive at 3X months or more.

Worst-case scenario: If 100 people had exactly the same type and stage of cancer as you, the 10 who did the worst　would have a high risk of dying within X/6 months.
